# Disability, quality of life, productivity impairment and employer costs of migraine in the workplace

**DOI:** 10.1186/s10194-021-01243-5

**Published:** 2021-04-21

**Authors:** Toshihiko Shimizu, Fumihiko Sakai, Hitoshi Miyake, Tomofumi Sone, Mitsuhiro Sato, Satoshi Tanabe, Yasuhiro Azuma, David W. Dodick

**Affiliations:** 1 Department of Neurology, Keio University School of Medicine, 35 Shinanomachi, Shinjuku-ku, Tokyo, 160-8582 Japan; 2 The Saitama International Headache Center, 6-11-1 Honmachi-Higashi, Chuo-ku, Saitama, 338-8577 Japan; 3 Corporate Executive Officer VP, Head of Health Promotion Unit at Fujitsu Co. Ltd, 4-1-1 Kamikodanaka , Nakahara-ku, Kawasaki, 211-8588 Japan; 4 National Institute of Public Health, 2-3-6 Minami, Wako-shi, Saitama, 351-0197 Japan; 5 Fujitsu General Limited, 3-3-17 Suenaga, Takatsu-ku, Kawasaki, 213-8502 Japan; 6 Health Promotion Unit at Fujitsu Co. Ltd, 4-1-1 Kamikodanaka, Nakahara-ku, Kawasaki, 211-8588 Japan; 7 Mayo Clinic College of Medicine, 13400 E Shea Blvd, Scottsdale, AZ USA

**Keywords:** Migraine, Prevalence, Disability, Impact, Economic loss, Presenteeism, Absenteeism, Workplace, Stigma, Work productivity

## Abstract

**Background:**

Migraine is the leading cause of days lost due to disability in the world among people less than 50 years of age. There is a paucity of evidence on the impact of migraine and other headache disorders and the cost and productivity losses in the workplace.

**Methods:**

Employee population survey assessed prevalence, characteristics, and disability of headache disorders at a Japanese information technology company. This study was supported by the World Health Organization Western Pacific Region Office and International Headache Society.

**Results:**

2458 (1963men, 495 women) out of 2494 responded to the survey that utilized ICHD-3 beta criteria. Among these, 13% (205 male/123 female) had migraine (M), 53% (1093 male/207 female) had tension-type headache (TTH) and 4% (61 male/27 female) had migraine and TTH (M/TTH). The number of days when productivity at work was reduced by half or more because of headache was significantly higher in migraine compared to TTH. The norm-based scoring of SF-12v2 was significantly lower in M/TTH and M than TTH. The economic loss due to absenteeism for migraine was calculated to be $ 238.3US$/year/person for day-off and 90.2US$/year/person for half-day off using migraine disability assessment score (MIDAS). The economic loss due to presenteeism for migraine was calculated to be $ 375.4US$/year/person using MIDAS and 2217US$/year/person using work productivity and activity impairment questionnaire (WPAI). Furthermore, estimated cost of productivity loss associated with presenteeism using WPAI was calculated at 21.3 billion US$/year in Japan as a whole.

**Conclusions:**

This study revealed a high prevalence and disease burden among employees with migraine that is associated with substantial losses in productivity and employer cost. These results support the development and implementation of workplace programs to improve migraine management in the workplace and reduce the burden and costs associated with lost workplace productivity.

**Supplementary Information:**

The online version contains supplementary material available at 10.1186/s10194-021-01243-5.

## Introduction

Headache disorders are a public health concern due to their high prevalence, disability and financial cost to society [1]. The World Health Organization estimates that the three most prevalent neurologic disorders worldwide are tension-type headache (1.5 billion), migraine (958.8 million) and medication overuse headache (58.5 million) [2]. The Global Burden of Diseases, Injuries, and Risk Factors study identified migraine as one of the 10 most disabling medical disorders in the world and the second leading cause of global neurological disease burden [3, 4]. In Asian and Oceanian countries, the importance of better headache care is being recognized as an important aspect of public health. Headache disorders are associated with a personal and social burden of pain, disability, impaired quality of life and financial cost. The estimates of the financial cost to society from lost work hours and reduced productivity are massive. Migraine is estimated to affect 8 million people in Japan and to cost the Japanese economy, in lost productivity, US$ 3 billion every year [5].

Despite the prevalence and disability associated with migraine, many may be suffering in silence at work, resulting in loss of significant productivity in Asian countries with growing economies [6]. In fact, population-based studies in North America demonstrated that presenteeism, which is working while sick, leads to more lost work time than absenteeism [7]. Approximately one-third of migraine attacks occur on workdays and two-thirds of these attacks result in a substantial loss of productivity [8]. Individuals with chronic migraine (> 15 headache days per month) experience four times more productive time lost compared to those with less frequent migraine attacks [9]. Determining the prevalence of presenteeism and absenteeism due to headache disorders in the growing economies of Asia is vital. Identifying and addressing factors that trigger attacks, providing education on self-management options, and providing access to standard of care treatments will help those affected better manage their condition, improve their ability to get to work or stay at work, and improve their function while at work.

To initiate a public health approach to headache disorders, we deployed a research survey on prevalence rates and disease burden associated with headache in the workplace. We focused on an Information Technology (IT) company in conducting this research. Workers of IT companies have been engaging in intellectual and cognitively challenging work and are considered to be a suitable population for our initial study on the impact on work productivity as a result of headache disorders. Because cognitive impairment has been shown to be a major source of disability associated with headache attacks, work efficiency is likely to be impaired by headache [10]. In addition, we believe that quantitative assessment of the reduction in work productivity and presenteeism due to headache should be possible in this homogeneous population.

## Methods

### Study design and population

This is a cross-sectional survey of workers of IT companies in Asia. Japan, Republic of Korea and the Philippines participated and conducted the study individually with the support of the World Health Organization Regional Office for the Western Pacific (WHO-WPRO) and the International Headache Society (IHS). Studies in these three countries followed the same protocol accredited by the WHO-WPRO. Since survey and data analysis are performed in each country, this study will summarize the findings in Japan.

The survey in Japan was conducted between May and September 2018 at Fujitsu with the support of the company’s Health Promotion Headquarters using tablet terminals for internal communications owned by all employees. The questionnaire survey was conducted only on the employees who gave their informed consent on the tablet screen.

The participants in these studies were given a comprehensive questionnaire to determine the prevalence of and the disability caused by migraine and tension-type headache divided into three parts. The first section of the questionnaire consisted of sociodemographic questions, the second part contained questions about health-related quality of life (HRQoL). The third part included questions pertaining to the diagnosis of headache, disease disability and productive loss due to headache. We also inquired about the reciprocal impact of headache on the work environment and the work environment on headache. Furthermore, we examined the health care utilization for headache and the reasons for consulting or not consulting among those reporting recurrent headache. We evaluated how to individuals had and are currently managing their headaches.

For the classification of headaches, questions were prepared based on the diagnostic criteria of the International Classification of Headache Disorders (ICHD-3 beta) [11]. In this study, ICHD-3 beta criteria were used and according to the criteria for medication overuse headache, “Patients with a pre-existing primary headache who in association with medication overuse develop a new type of headache or a marked worsening of their pre-existing headache that, in either case, meets the criteria for 8.2 Medication-overuse headache (or one of its subtypes) should be given both this diagnosis and the diagnosis of the pre-existing headache”. Because we could not be certain from the questionnaires used whether a new type of headache or a marked worsening of a pre-existing headache occurred, we restricted the analysis to the primary headache disorder type for each subject.

The 12-item short-form health survey second edition (SF-12v2) Japanese version was used for analysis of HRQoL [12, 13]. Using the SF-12v2 Japanese version of the scoring program by iHope International, norm-based scoring was calculated for eight subscales of SF-12v2. The subscales are: Physical functioning (PF), daily role function (body, Role physical; RP), body pain (Bodily pain; BP), general health (GH), vitality (Vitality; VT), society Life function (Social functioning; SF), daily role function (Mental, Role emotional; RE), mental health (Mental health; MH).

Furthermore, questions related to the migraine disability assessment score (MIDAS) [14, 15] and Work Productivity and Activity Impairment questionnaire (WPAI) were used to measure the severity of impact on work and daily life [16].

To estimate the economic loss due to headache, we calculated the number of days of headache, moderate or severe headache, the days off work due to headache, the half-days off work due to headache, and when work efficiency was reduced to less than half due to headache, using MIDAS. In addition, the degree to which the headache impacted work productivity was also evaluated on the WPAI questionnaire. We asked the participants whether they had the symptoms related to headache disorder on days when headaches are not experienced.

The economic loss due to absenteeism and presenteeism caused by headache was calculated using the age-specific wage in the IT industry according to the Basic Survey on Wage Structure in 2018 by the Ministry of Health, Labor and Welfare, since this survey was conducted in 2018 [17].

Economic losses due to absenteeism were calculated for a full day-off and half-day off. The number of day-off multiplied by the daily wage and the number of half-days off multiplied by 50% of the daily wage, and these were converted to annual amounts.

The economic loss due to presenteeism was calculated by multiplying the number of the days when work productivity was reduced to less than half from MIDAS by wage multiplied by 0.5 and converted to annual amount. Furthermore, the impact of WPAI on work productivity was converted into a ratio. This ratio, the number of days worked with headache and daily wage were multiplied, and the result was converted to annual monetary value. We considered this as the economic loss due to presentism estimated by WPAI. Because of the absence of information on the degree of productivity loss per each day of presenteeism, we assumed that work efficiency was halved, when calculating presenteeism economic losses from MIDAS data. Also, we did not ask WPAI for each headache and the value is the mean of headache of each subject. Therefore, when calculating the amount of economic loss due to presenteeism from WPAI data, we assumed that the degree of work efficiency decline was the same on days with headaches. Hence, the economic loss due to MIDAS may be underestimated, while the economic loss due to WPAI may be overestimated, and the economic loss from presenteeism in migraine in Japan is estimated to be at least the value calculated by MIDAS and up to the value calculated by WPAI. The conversion from Japanese yen to US dollar was calculated at the conversion rate of 112 yen to one US dollar.

To estimate the effects of headache on work environment and work statistically, the answers to the questions, “Always”, “Often”, “Sometimes”, “Rarely” and “Never”, were scored as 5, 4, 3, 2, and 1, respectively, and we analyzed these results using these scores.

### Statistical methods

According to the ICHD-3 beta included in the questionnaire of this study, the group was classified into five groups: a group that satisfies the diagnostic criteria for migraine (migraine group, migraine; M), a group that satisfies the diagnostic criteria for tension-type headache (tension-type headache group, TTH), a group that satisfies the diagnostic criteria for migraine and tension-type headache (M/TTH), a group that does not meet the diagnostic criteria for migraine or tension-type headache (HA) and a group that does not have headache (no headache; NHA).

In the questionnaire survey of this study, the age of the participants was between 22 and 66 years old and did not include the younger and older participants. Therefore, we used this result as the prevalence of headache at working age in Japan for the subsequent analysis in this study.

Descriptive statistical analyses were performed to compare clinical features and HRQoL among these five groups. Disease burden and the status of medical consultation were compared among M, TTH, M/TTH and HA.

For comparison between multiple groups, a post-hoc test by the Bonferroni was performed after one-way ANOVA. They are; age, body mass index (BMI), HRQoL, disease disability, impact of headache, economic loss due to headache and the effects of headache on work environment and work.

Frequency data such as working hours and overtime hours, the symptoms related to headache on days even if headache does not occur and consultation to the medical institutions was subjected to residual analysis after chi-square test. For residual analysis, cut-off values of adjusted residuals was set at ≤ − 2 or ≥ 2.

Logistic regression was conducted using the forced entry method in order to search for migraine-inducing factors with the dependent variable as the trigger of migraine or tension-type headache. As explanatory variables, age, gender, sleep time, overtime hours, computer hours, lots of work, quota achievement, lack of meals, lack of water intake, and drinking were used. For all analyses, *p* < 0.05 were considered statistically significant.

All statistical results were analyzed based on non-missing data. Stata (release 15; Light Stone) was used for analysis.

## Results

### Responses of participants

We received 2494 responses from the questionnaire. The analysis was performed on 2458 cases excluding 36 cases lacking the description overtime hours and missing demographic information on age and gender.

### Headache classification and their characteristics

Among 2458 cases, migraine group (M) accounted for 13% and tension-type headache group (TTH) for 53%, and migraine and tension-type headache group (M/ TTH) is 4% (88 cases, 61 men 3.1%, 27 women 5.5%) and other headache groups (Headache other than migraine and / or tension-type headache; HA) 15% and 15% (no headache; NHA) group as shown in Table 1. Statistically, the mean age of M and HA was significantly younger than TTH (*p* < 0.001, one-way ANOVA followed by Bonferroni’s post hoc test). Also, BMI of M was significantly lower than TTH (*p* = 0.049, one-way ANOVA followed by Bonferroni’s post hoc test). There was no significant difference in the working hours for 1 week and overtime hours for 1 month (supplementary material; Table 1).

**Table 1:** Headache classification

	M	TTH	M/TTH	HA	NHA	Total	*p*-value
Number, n (%)	328 (13)	1300 (53)	88 (4)	360 (15)	382 (15)	2458	
male, n	205	1093	61	265	339	1963	
female, n	123	207	27	95	43	495	
Age (years), mean (SD)	44.5 (8.6)	46.9 (8.7)	45.5 (8.3)	44.2 (9.6)	46.0 (10.8)		< 0.001^a^
BMI (kg/m^2^), mean (SD)	23.0 (3.7)	23.7 (3.7)	23.4 (3.7)	23.2 (3.5)	23.5 (3.6)		0.049^a^

Continuous variables are reported as mean (standard deviation [SD]) for non-missing observations

*M* migraine group, *TTH* tension-type headache group, *M/TTH* a group that satisfies the diagnostic criteria for migraine and tension-type headache, *HA* a group that does not meet the diagnostic criteria for migraine or tension-type headache, *NHA* a group that does not have headache, *BMI* body mass index

^a^One-way ANOVA

### HRQoL

Table 2 shows the norm-based scoring of the SF-12v2 Japanese version. In group comparison, RP, BP, VT, SF, RE and MH scores were significantly lower in M and M/TTH than TTH and NHA (*p* < 0.05, one-way ANOVA followed by Bonferroni’s post hoc test). In addition, GH was significantly lower in M/TTH than NHA and in M than TTH and NHA (*p* < 0.05, one-way ANOVA followed by Bonferroni’s post hoc test).

**Table 2:** Norm-based scoring of SF-12v2

	M	TTH	M/TTH	HA	NHA	*p*-value^a^
Physical functioning, mean (SD)	50 (10)	51 (10)	49 (11)	50 (10)	51 (10)	0.472
Role physical, mean (SD)	46 (11)	49 (10)	45 (11)	47 (11)	50 (9)	< 0.001
Bodily pain, mean (SD)	43 (11)	50 (10)	45 (10)	47 (11)	52 (11)	< 0.001
General health, mean (SD)	48 (10)	51 (8)	48 (9)	49 (10)	53 (9)	< 0.001
Vitality, mean (SD)	46 (9)	48 (8)	44 (10)	47 (8)	50 (9)	< 0.001
Social functioning, mean (SD)	49 (10)	51 (9)	48 (10)	50 (10)	51 (9)	< 0.001
Role emotional, mean (SD)	43 (11)	47 (10)	44 (11)	44 (11)	50 (9)	< 0.001
Mental health, mean (SD)	44 (10)	48 (9)	44 (10)	45 (10)	49 (10)	< 0.001

Continuous variables are reported as mean (SD) for non-missing observations

*M* migraine group, *TTH* tension-type headache group, *M/TTH* a group that satisfies the diagnostic criteria for migraine and tension-type headache, *HA* a group that does not meet the diagnostic criteria for migraine or tension-type headache, *NHA* a group that does not have headache

^a^One-way ANOVA

### Impact of headache on work productivity

In the past 3 months, the average number of headache days and the number of moderate or severe headache days were significantly higher for M, M/TTH and HA than for TTH (*p* < 0.05, one-way ANOVA followed by Bonferroni’s post hoc test, Table 3). The average number of days off for 1 day or half day due to headache in the past 3 months did not differ significantly between each headache group (Table 3). On the other hand, the number of the days when work efficiency was reduced to less than half due to headache were significantly higher in M and M/TTH than in TTH (*p* < 0.05, one-way ANOVA followed by Bonferroni’s post hoc test, Table 3). The impact of headache on work productivity was evaluated in 10 steps using WPAI and significantly higher in M/TTH, M, HA compared to TTH (*p* < 0.001, one-way ANOVA followed by Bonferroni’s post hoc test, Table 3).

**Table 3:** Number of headache days and working days affected by headache in 3 months, and the impact of headaches on work productivity by WPAI

	M	TTH	M/TTH	HA	*p*-value^a^
Headache (days), mean (SD)	5.5 (8.4)	2.4 (6.5)	6.4 (12.3)	3.9 (10.4)	< 0.001
Severe Headache (days), mean (SD)	1.9 (3.6)	0.5 (2.4)	1.6 (2.6)	1.1 (4.6)	< 0.001
One day off (days), mean (SD)	0.3 (0.9)	0.1 (1.2)	0.2 (0.5)	0.1 (0.6)	0.084
Half day off (days), mean (SD)	0.2 (1.6)	0.1 (0.9)	0.1 (0.4)	0.1 (0.5)	0.081
Reduced productivity (days), mean (SD)	1.0 (2.4)	0.2 (1.3)	0.8 (2.1)	0.5 (2.3)	< 0.001
The impact of headaches on work productivity					
WPAI score, mean (SD)	4.7 (2.7)	2.0 (2.1)	4.1 (2.8)	3.5 (2.6)	< 0.001

Continuous variables are reported as mean (SD) for non-missing observations

*M* migraine group, *TTH* tension-type headache group, *M/TTH* a group that satisfies the diagnostic criteria for migraine and tension-type headache, *HA* a group that does not meet the diagnostic criteria for migraine or tension-type headache, *WPAI* work productivity and activity impairment questionnaire

^a^One-way ANOVA

The annual economic loss per person due to absenteeism was higher in M compared to M/TTH, TTH, HA for day off and half day off holidays, but showed no significant difference (Table 4). The annual economic loss per person due to presenteeism calculated by the results of MIDAS and WPAI were significantly higher in M and M/TTH than in TTH (*p* < 0.01, one-way ANOVA followed by Bonferroni’s post hoc test, Table 4).

**Table 4:** Annual economic loss per person due to absenteeism and presenteeism

	M	TTH	M/TTH	HA	*p*-value^a^
Absenteeism (calculated by MIDAS)					
Day off (US$), mean (SD)	238.3 (739.9)	82.9 (1142)	153.9 (409.2)	123.0 (511.0)	0.080
Half day off (US$), mean (SD)	90.2 (462.7)	30.1 (431.3)	40.2 (163.7)	47.8 (209.3)	0.111
Presenteeism					
Calculated by MIDAS (US$), mean (SD)	375.4 (1039)	70.9 (533.8)	324.1 (878.1)	191.1 (967.1)	< 0.001
Calculated by WPAI (US$), mean (SD)	2217 (4497)	562.1 (266)	2621 (6077)	1267 (4356)	< 0.001

Continuous variables are reported as mean (SD) for non-missing observations

*M* migraine group, *TTH* tension-type headache group, *M/TTH* a group that satisfies the diagnostic criteria for migraine and tension-type headache, *HA* a group that does not meet the diagnostic criteria for migraine or tension-type headache, *MIDAS* migraine disability assessment score, *WPAI* work productivity and activity impairment questionnaire

^a^One-way ANOVA

**Fig. 1:**
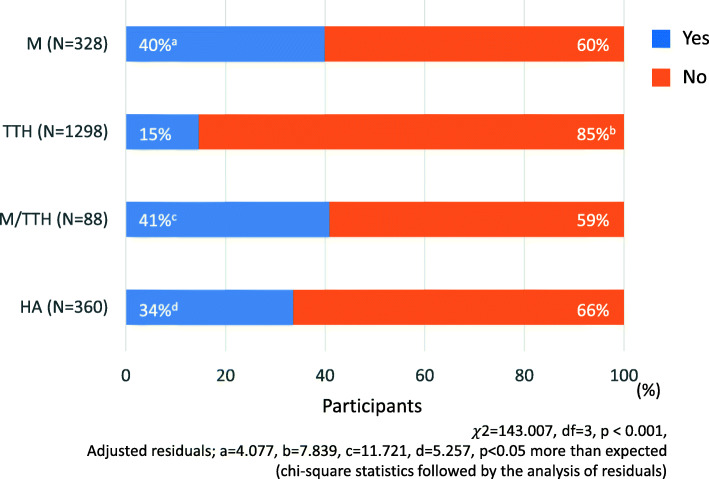
Symptoms related to headache disorder on days when headaches are not experienced. M, migraine group; TTH, tension-type headache group; M/TTH, a group that satisfies the diagnostic criteria for migraine and tension-type headache; HA, a group that does not meet the diagnostic criteria for migraine or tension-type headache

In addition, symptoms related to headache on days even if headache does not occur were observed in 40% of M, 15% of TTH, 41% of M/TTH and 34% of HA, and they were significantly higher than expected in M/TTH, M, HA (*p* < 0.05, chi-square statistics followed by the analysis of residuals, Fig. 1). These symptoms included anxiety, depressive state, difficult to concentrate and sensitive to light (supplementary material; Table 2). Among these symptoms, difficulty to concentrate, feeling tired and stiff shoulders were frequently observed.

Effects of headache on work environment and work were shown in Table 5. M, M/TTH and HA showed significantly higher values compared to TTH for lack of understanding of headaches in the workplace, impaired relationships due to headaches and burdening bosses and colleagues with headaches (*p* < 0.01, one-way ANOVA followed by Bonferroni’s post hoc test). Furthermore, M and M/TTH showed significantly higher values compared to TTH and HA to lose their energy to work and not to concentrate on work due to headache (*p* < 0.01, one-way ANOVA followed by Bonferroni’s post hoc test).

**Table 5:** Effects of headache on work environment and work

	M	TTH	M/TTH	HA	*p*-value^a^
Lack of understanding, mean (SD)	2.0 (1.2)	1.4 (0.8)	2.0 (1.1)	1.7 (1.1)	< 0.001
Have difficulty with human relations, mean (SD)	1.5 (0.8)	1.2 (0.5)	1.4 (0.8)	1.3 (0.7)	< 0.001
Burden on others, mean (SD)	1.6 (0.8)	1.2 (0.5)	1.5 (0.8)	1.4 (9.8)	< 0.001
Lack energy, mean (SD)	2.8 (1.0)	1.8 (9.9)	2.7 (0.9)	2.3 (1.0)	< 0.001
Not able to concentrate, mean (SD)	2.8 (1.0)	1.9 (0.9)	2.8 (0.9)	2.3(1.0)	< 0.001

Continuous variables are reported as mean (SD) for non-missing observations

*M* migraine group, *TTH* tension-type headache group, *M/TTH* a group that satisfies the diagnostic criteria for migraine and tension-type headache, *HA* a group that does not meet the diagnostic criteria for migraine or tension-type headache

^a^One-way ANOVA

In logistic regression with tension-type headache as a control, age, gender, lack of sleep, completion of work and skipping meals were correlated significantly with occurrence of migraine (*p* < 0.05), and it is suggested that these may be factors associated with migraine (Table 6).

**Table 6:** Factors that may be associated with migraine

	Odds Ratio	Std. Err.	z	*P* > |z|	95% CI
Age	0.9791165	0.0073349	−2.82	0.005	0.9648455–0.9935987
Gender	0.3635942	0.0525101	−7.01	0	0.2739598–0.4825553
Lack of sleep	2.045667	0.2924829	5.01	0	1.545728–2.707304
Overtime work	0.9279385	0.1740869	−0.4	0.69	0.6424341–1.340324
Long time PC use	1.237306	0.2033557	1.3	0.195	0.8965589–1.707557
Too much work	0.984432	0.188815	−0.08	0.935	0.6759654–1.433663
Completed project	2.14913	0.7591344	2.17	0.03	1.075459–4.294687
Skipping meal	1.730537	0.4820394	1.97	0.049	1.002489–2.987324
Dehydration	1.161006	0.2004847	0.86	0.387	0.8276512–1.628627
Alcohol intake	1.198216	0.184249	1.18	0.24	0.8864343–1.619661

*CI* confidence interval, *Std. Err* standered error, *PC* personal computer

### Consultation to the medical institutions and treatment for headache

**Fig. 2:**
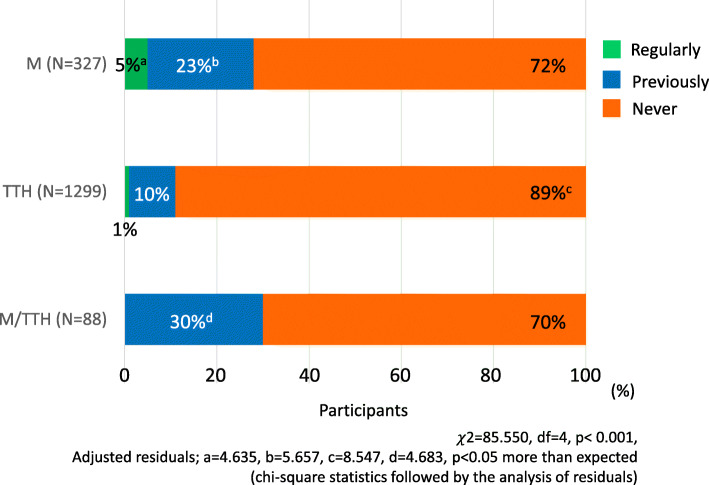
Consultation to the medical institutions. M, migraine group; TTH, tension-type headache group; M/TTH, a group that satisfies the diagnostic criteria for migraine and tension-type headache

As shown in Fig. 2, 5 % of M answered that they “consult regularly”, 1% of TTH, and none of M/TTH. “Previously visited the hospital (now discontinued)” was 23% for M, 10% for TTH, and 30% for M/TTH, and this was significantly higher than expected in M and M/TTH (*p* < 0.001, chi-square statistics followed by the analysis of residuals). “Never visited the medical institution” was 72% for M, 89% for TTH, and 70% for M/TTH. In addition, only 5% of M patients were prescribed acute medicines and 2% were prescribed preventive drugs.

The most common reason for not seeing a medical institution was because they did not consider their headaches severe enough to require a consultation (57% of M, 76% of TTH and 61% of M/TTH). However, only 35% of M, 21% of TTH and 41% of M/TTH thought they could manage their own headache.

As for the self-care of headache, “lying down” was 66% in M, 41% in TTH, 59% in M/TTH, and “use over the counter” was 67% in M, 36% in TTH and 69% in M/TTH. “Lying down” and “use over the counter” were significantly higher than expected in M and M/TTH (*p* < 0.001, chi-square statistics followed by the analysis of residuals).

## Discussion

This study revealed a high prevalence and disease burden among employees with migraine that is associated with substantial losses in productivity and employer cost. Amongst the 2458 respondents (98.5% of those surveyed) 17% had migraine, and compared to individuals with no headache or tension-type headache, people with migraine had significantly more missed workdays, and experienced a greater impact on work productivity, physical and mental health and economic cost to the employer.

Presenteeism is defined as an individual’s loss of work productivity due to health conditions and the symptoms of a disease. In a study involving 7959 employees evaluated over a 4-year period, 22 health conditions were studied for their effect on daily productivity of employees at a large health care system. The conditions with the highest estimated daily productivity loss and annual cost per person were chronic back pain, mental illness, general anxiety, migraine or severe headaches, neck pain, and depression. Allergies and migraine or severe headaches had the highest estimated annual company cost [18]. Their result indicates that 16% of workforce presenteeism may be due to migraine with a cost of US $240 billion dollars per year. Furthermore, according to the Japan National Health and Wellness Survey of pooled commercial data, migraine patients have been reported to experience significantly higher presenteeism than controls in Japan [19].

Our study focused on migraine and demonstrated that presenteeism causes more economic loss than absenteeism. Based on our findings, we can predict the annual economic loss due to migraine in Japan as a whole. The age range of respondents in this study was 25 and 65 and according to statistics from the Statistics Bureau of the Ministry of Internal Affairs and Communications, the number of working people between the ages of 25 and 65 in Japan in 2018 is 52,400,000 [20]. Using economic loss due to migraine and working population data from the Statistics Bureau of Japan, the annual economic loss due to presenteeism is US $3.3 billion, calculated from the number of days when work efficiency has fallen to less than half due to headaches using MIDAS. On the other hand, the annual economic loss due to presenteeism using WPAI is estimated to be US $21.3 billion. In addition, the annual economic loss due to absenteeism estimated by MIDAS was calculated to be US $2.7 billion in total, with the loss due to one day off being US $2.0 billion and the loss due to half day off being US $0.7 billion.

Although migraine is classically described as a chronic disease with paroxysmal or episodic manifestations, the presence and burden of persistent symptoms between attacks, including photophobia and cognitive dysfunction, has been reported [21]. An anti-CGRP antibody recently approved by the FDA in the United States has been reported to improve these interictal symptoms [22]. Our questionnaire also showed that 40% of those with migraine have interictal symptoms, and the impact of these symptoms on work was significantly higher in the migraine compared to the tension-type headache group. This suggests that migraine may have an impact on work efficiency and economic losses even on days without headache. In addition, our questionnaire survey revealed that 98% of migraine sufferers had never been treated with preventive medications. It has also been reported that many migraine patients use over-the-counter instead of prescription medication in East Asia [23]. Therefore, in order to improve the economic loss due to migraine, it is important to improve awareness around the availability and importance of using preventive treatments that reduce attack frequency and improve interictal burden in those patients with an unmet treatment need.

The impact of migraine in the workplace has also been correlated with the frequency of days with headache [24, 25]. For those with frequent headache (10–14 days per month), the estimated number of days per year missing from work is 2 days while there is 46 days with reduced productivity (presenteeism). This loss in productivity accounts for approximately 20% of the work year. Higher numbers for missed and lost productive days per year for those with more than 15 headache days per month are 3.5 days and 87 days respectively, accounting for 38% of the work year [24]. Furthermore, it is reported that patients with chronic migraine experienced greater impairment and less productivity than those with episodic migraine, according to WPAI scores in Japan [26]. Our data also illustrates that the number of days with presenteeism was significantly higher in chronic migraine compared with episodic migraine.

A recent study assessed the impact of a migraine care support program offered by a healthcare company as a complimentary service to medical care for its Swiss based employees and their family members living with migraine [27]. The study results demonstrated that educational and counseling support program within an employee population can significantly decrease migraine-related disability and promote disease self-management among employees. Fujitsu, the participating company of this study, has initiated an employee migraine wellness program with GPAC (Global Patient Advocacy Coalition) of the International Headache Society [28].

Stigma is an established construct in the social sciences that describes a characteristic, trait, or diagnosis that is used to discredit an individual and leads to prejudice, discrimination, and loss of status [29]. The stigma faced by employees with migraine is substantial, and like in our study, stigma contributes to the burden and economic loss due to migraine. In this study, individuals with migraine reported a significant lack of understanding of headache in the workplace, impaired relationships due to headache, and guilt about burdening bosses and colleagues with headaches. In addition, the most common reason for not seeking medical consultation was that they did not consider their headache disorder severe enough to require a consultation. Stigma and lack of awareness of burden of migraine may well explain our data that 72% of individuals did not seek medical consultation for migraine despite the fact that 63% of patients indicated that they could not cope with their illness.

Our results are consistent with the stigmatizing attitudes of those who surround people with migraine. Using the Stigma Scale for Chronic Migraine (SSCI), stigma associated with chronic migraine has been found to be higher than other neurological diseases including stroke, epilepsy, multiple sclerosis, Parkinson’s disease, motor neuron disease, and epilepsy [30, 31]. In a recent study, more than 40% of people who knew at least one person with migraine felt that people with migraine use their illness as an excuse to avoid family, work, or school commitments and/or exaggerate their symptoms. More than one third (36%) believed that someone’s migraine attacks are caused by their own unhealthy behavior and approximately one-third of people believed those with migraine make things difficult for their co-workers (29%) [30, 32]. These attitudes and beliefs were consistent among all individuals surrounding a person with migraine including co-workers, friends, and family members. Employers also harbor stigma toward those with migraine. Only 22% consider migraine to be a “serious enough reason for an employee to be absent from work”, lower than for any other reason, including depression, anxiety, stress, the flu, or the common cold [33]. These data highlight the importance of ensuring that any disease-related education with a workforce also target people without migraine to create a better understanding of the prevalence and impact of this disease on their co-workers.

There are several limitations that should be noted in this study. This research is conducted through the company’s tablet terminal. Since all employees have terminals, there is no restriction on access to the questionnaire. However, since participation in the questionnaire was voluntary, ascertainment bias is a risk as only those with an interest in headache, especially those affected, would participate. For this reason, questions regarding daily quality of life were included so that people without headache may have been more interested to participate. In addition, the diagnosis of migraine was not confirmed by a physician. However, the diagnostic portion of the questionnaire included all criteria from ICHD-3 beta, and this methodology is commonly used in population-based studies of headache and migraine.

Despite these limitations, the high prevalence and disease burden among employees with migraine in this study provide the basis and a mandate for measures designed to screen employees for disabling headache and provide appropriate education and care. The substantial losses in productivity and high employer costs also support the development and implementation of workplace programs to raise awareness and understanding, reduce stigma, improve migraine management and reduce the burden and costs associated with lost workplace productivity.

## Conclusions

This employee population survey revealed a high prevalence and disease burden of migraine that is associated with substantial losses in productivity and employer cost. In employees with migraine, presenteeism causes more economic loss than absenteeism. These results support the development and implementation of workplace programs to improve migraine management in the workplace and reduce the burden and costs associated with lost workplace productivity.

## Supplementary Information

**Additional file 1: Table S1.** Working hours/ week and overtime working hours/month.

**Additional file 2: Table S2.** Symptoms related to headache on days when headaches are not experienced.

## Data Availability

The datasets used and/or analyzed during the current study are available from the corresponding author on reasonable request.

## Abbreviations

ANOVA: Analysis of variance
BMI: Body mass index
BP: Bodily pain
CGRP: Calcitonin gene-related peptide
FDA: Food and drug administration
GH: General health
GPAC: Global patient advocacy coalition
HA: Headache
HRQoL: Health-related quality of life
ICHD-3 beta: International classification of headache disorders 3rd edition beta version
IHS: International headache society
IT: Information technology
M: Migraine
MIDAS: Migraine Disability Assessment Score
MH: Mental health
M/TTH: Migraine and tension-type headache
NHA: No headache
PF: Physical functioning
RE: Role emotional
RP: Role physical
SD: Standard deviation
SF: Social functioning
SF-12v.2: The 12-item short-form health survey second edition
SF-36: The 36-item short-form health survey
TTH: Tension-type headache
VT: Vitality
WHO: World Health Organization
WHO-WPRO: World Health Organization Regional Office for the Western Pacific
WPAI: Work Productivity and Activity Impairment Questionnaire
